# Injection Laryngoplasty Using Autologous Fat Enriched with Adipose-Derived Regenerative Stem Cells: A Safe Therapeutic Option for the Functional Reconstruction of the Glottal Gap after Unilateral Vocal Fold Paralysis

**DOI:** 10.1155/2018/8917913

**Published:** 2018-04-15

**Authors:** José M. Lasso, Daniel Poletti, Batolomé Scola, Pedro Gómez-Vilda, Ana I. García-Martín, María Eugenia Fernández-Santos

**Affiliations:** ^1^Plastic and Reconstructive Surgery, Hospital General Universitario Gregorio Marañón, Instituto de Investigación Sanitaria Gregorio Marañón (IISGM), Universidad Complutense (UCM), Madrid, Spain; ^2^ORL, Hospital General Universitario Gregorio Marañón, Madrid, Spain; ^3^Neuromorphic Processing Lab, Center for Biomedical Technology, Universidad Politécnica de Madrid, Madrid, Spain; ^4^Nursery, Hospital General Universitario Gregorio Marañón, Madrid, Spain; ^5^Cell Therapy Unit, Instituto de Investigación Sanitaria Gregorio Marañón (IISGM), Red de Terapia Celular (TERCEL), Hospital General Universitario Gregorio Marañón, Madrid, Spain

## Abstract

**Background:**

Paralysis of one vocal fold leads to glottal gap and vocal fold insufficiency that has significant impact upon a patient's quality of life. Fillers have been tested to perform intracordal injections, but they do not provide perdurable results. Early data suggest that enriching fat grafts with adipose-derived regenerative cells (ADRCs) promote angiogenesis and modulate the immune response, improving graft survival. The aim of this study is to propose ADRC-enriched adipose tissue grafts as effective filler for the paralyzed vocal fold to use it for functional reconstruction of the glottal gap.

**Method:**

This is the first phase I-IIA clinical trial (phase I/IIA clinical trial, unicentric, randomized, controlled, and two parallel groups), to evaluate the safety of a new therapy with ADRC-enriched fat grafting (ADRC: group I) for laryngoplasty after unilateral vocal fold paralysis. Control group patients received centrifuged autologous fat (CAF: group II) grafts. Overall mean age is 52.49 ± 16.60 years. Group I (ADRC): 7 patients (3 males and 4 females), 52.28 ± 20.95 year. Group II (CAF): 7 patients (3 males and 4 females), 52.71 ± 12.59 year.

**Results:**

VHI-10 test showed that preoperative mean score was 24.21 ± 8.28. Postoperative mean score was 6.71 ± 6.75. Preoperative result in group I was 21.14 ± 3.58 and postoperative result was 3.14 ± 3.53. Preoperative result for group II was 27.29 ± 10.66. Postoperative score in group II was 10.29 ± 7.52. Wilcoxon and the Student *t*-tests showed that the patient's self-perception of posttreatment improvement is larger when ADRCs are used. Comparing pre- and posttreatment voice quality analysis, group I showed a *p* = 0.053. Group II showed a *p* = 0.007. There would be no significant differentiation between pre- and posttreatment results. This is true for group II and limited for group I.

**Conclusions:**

This prospective trial demonstrates the safety and efficacy of the treatment of glottal gap defects utilizing ADRC-enriched fat grafts. This trial is registered with NCT02904824.

## 1. Introduction

Paralysis of one of the vocal folds (VF) may have a significant impact upon a patient's quality of life. The affected patient may present glottal insufficiency which leads to poor breathy voice, problems with their swallowing risking possible aspiration, a weak cough, and the sensation of breath shortness [1].

Unilateral vocal fold paralysis (UVFP) is one of the main causes of glottal gap (GG) and VF insufficiency. UVFP occurs from a dysfunction of the recurrent laryngeal or vagus nerve innervating the larynx, arising from a variety of causes like iatrogenic injury, most commonly to the recurrent laryngeal nerve, but it can be secondary to viral infection or direct trauma from surgery (thyroidectomy, carotid endarterectomy, skull base surgery, anterior cervical spine surgery, thoracic, or mediastinal surgery among them).

Depending on the type of paralysis (bilateral, unilateral, in abduction, or adduction), the treatment may be different, consisting on expectancy with or without phoniatric rehabilitation or surgery. Surgery of UVFP consists basically in two techniques: an open surgery on the laryngeal box or an injection laryngoplasty into the VF with refilling materials [2]. Furthermore, open surgery requires a major surgical operation and can be associated with significant morbidity in addition to an extensive cost to the healthcare system [3].

In the last decade, several fillers have been tested to perform intracordal injections; these “injectable implants” present high viscosity, consisting of particles or even cells, collagen, hyaluronic acid, or autologous fat among others. In UVFP, they are injected into the paralyzed VF to increase its volume and correct the GG. These procedures are considered effective when medialization of the affected VF allows a total contact on phonation and the physiological and biomechanical properties of the larynx have been restored. Nevertheless, they do not provide perdurable results, and patients usually need multiple injections. Given these challenges, researchers continue looking for novel substances to treat the GG.

Since autologous fat transfer was initially reported in 1893 [4], there is evidence demonstrating that it may be useful in the treatment of some anatomical defects; however, current methods of fat harvesting, processing, and delivery are still being standardized, which results in unpredictable graft survival and inconsistent outcomes [5, 6].

Early data suggest that enriching fat grafts with supplemental adipose-derived regenerative cells (ADRCs) promote normal angiogenesis, decrease apoptosis, and modulate the immune response, each of which could improve graft survival [7]. Furthermore, the regenerative cells in adipose tissue are so abundant that the need for culture expansion to reach a therapeutic dose is eliminated. Thus, a patient's adipose tissue can be harvested, processed (in part to extract ADRCs), and injected back into the patient during the same surgical procedure. Different works report that they facilitate wound healing and angiogenesis [8–10], and this having also being shown in a recent clinical trial [11] in which ADRCs improved the fat graft survival in breast tissue. However, the “in vivo” growth and differentiation characteristics of stem cells remain unclear, and certain tumorigenic risks cannot be disregarded [12].

On the other hand, large prospective clinical trials have not been reported about the use of ADRC-enriched fat grafting for patients presenting GG after UVFP. As such, the aim of this study is to propose the use of ADRC-enriched adipose tissue grafts as effective filler for the paralyzed vocal fold in order to use it as a novel therapeutic option for the functional reconstruction of the GG.

## 2. Patients and Methods

The present study corresponds to the first phase I-IIA clinical trial (phase I/IIA clinical trial, unicentric, randomized, controlled, and two parallel groups), to evaluate the safety of a new therapy with ADRC-enriched fat grafting (AF + ADRCs) for VF laryngoplasty after UVFP originating a GG in the International Conference on Harmonization (ICH) E2F format. The control group patients were treated with centrifuged autologous fat (CAF) grafts. The reporting interval ranged from July 2012 to September 2014.

Ethics Committee approval was obtained at the institution where it was held, and all patients provided specific written informed consent.

Coprimary endpoints included the following:

- Safety of the injection of ADRCs into the VF
- Improvement in overall closure of the VF at least six months after index procedure

Secondary endpoints consisted of improvement in VF volume and quality of life in comparison to the use of centrifuged fat. A software application for voice quality analysis [13] was used to measure and compare the evolution of different voice parameters before surgery, 30 days and at least 180 days after the implant. In addition, adverse event profile and resource utilization were evaluated.

### 2.1. Patient Selection

Female and male patients (older than 18 years) presenting GG after UVFP were eligible for enrollment. Key inclusion criteria also included the absence of compensation of the GG from the contralateral VF, the ability to undergo abdomen liposuction for graft acquisition, and the absence of granulomae, tumors, or visual lesions by direct laryngoscopy in the affected VF. Major exclusion criteria were history of autoimmune disorder, active or chronic infectious diseases, or major surgeries 28 days before the VF surgery.

Patients were randomly distributed into 2 surgical groups to receive one of the following therapeutic strategies:

Group I VF infiltration of AF washed with Lactated Ringers solution using gravity sedimentation/floatation enriched with autologous ADRCs (AF + ADRCs)
Group II VF infiltration of AF centrifuged during 3 minutes at 3000 revolutions/minute (CAF)

### 2.2. Surgical Treatment

Surgery consisted in 2 steps: step 1, adipose tissue harvesting; step 2, infiltration of adipose tissue into the VF.

In between both procedures, the harvested fatty tissue was processed following two different protocols depending on the surgical group. These procedures were carried out by a biologist in a laboratory adjacent to the operation room, under sterile measures. The real effective time required for laboratory process in group I was longer than that in group II. In order to avoid bias coming from the surgical protocol, the time elapsed for processing the CAF was deliberately prolonged to the processing time in group I, before VF injection.

At least 180 cm^3^ of the abdominal fat was harvested using standard tumescent, syringe-based liposuction under local anesthesia, and sedation because this was the minimum volume of fat required by the device used for processing ADRCs.

### 2.3. Step I: Adipose Tissue Harvesting

Prior to lipo-harvesting, target areas were infiltrated with standard tumescent solution (Lactated Ringers, 1% lidocaine and 1 mg/mL epinephrine; 250 cm^3^). Through stab incisions at the umbilicus, adipose tissue was collected from the infra-abdominal area using a 3 mm Mercedes tip three-hole blunt cannula (Byron Medical Inc., Tucson, AZ) under low negative pressure.

After the first surgical procedure, patients were transported to a recovery room, to safely regain consciousness from sedation and receive appropriate postoperative care, until the second part of the procedure was performed.

### 2.4. Step II: Injection Laryngoplasty with AF + ADRCs or with CAF

Patients were transported again to the operation room. Under general anesthesia and orotracheal intubation, the VF were exposed and explored with a rigid direct suspension laryngoscope, and then transoral injection was performed under direct microscopic visualization.

A 10 cm^3^ syringe holding the fat graft (the aspect of it was identical in every patient, so the surgeon could not identify the surgical group of the patient) was brought to the operation room. The material was injected lateral to the vocal fold where it arises from the vocal process. This closed the middle and posterior gap between the VF. Eventually, a second injection was performed lateral to the midvocal fold to achieve a slight overmedialization. The injection was aimed deep into the thyroarytenoid muscle (a depth of 2-3 mm) using a 19-gauge syringe (Micro-France®, St. Aubin, France), to ideally achieve 20% to 30% bulging across the midline. After the injection, the vocal fold was stroked with a suction tube to smoothen the medial edge. Augmentation of the anterior third of the vocal fold was avoided.

After graft delivery, the patient was extubated and discharged to the recovery room.

### 2.5. Postoperative Care

Patients were discharged from the hospital on the same day or on the first postoperative day. All patients attended routine control visits 7, 30, and 180 days after surgery for general check and laryngeal assessment. After this period, checking was done every 180 days. Voice records were performed 30 and 180 days after the surgery.

### 2.6. Preparation of the CAF and ADRC-Enriched Fat Graft

In group I, adipose tissue was divided into two equal fractions, one for the extraction of ADRCs and the other for use as the fat graft. This preparation was done at the surgical laboratory. One fraction of the lipoaspirate was added to the Celution® System (Cytori Therapeutics, San Diego, CA) where the ADRCs were released from their bound matrix with the addition of a proteolytic enzyme reagent (Celase®, Cytori Therapeutics, San Diego, CA), washed to remove residual enzyme, and then concentrated within the closed automated system in the operating room. Upon completing this process (approximately 90 minutes), the suspension of ADRCs (~5 mL) was retrieved from the Celution System using an 18-gauge spinal needle. The second fraction of adipose tissue was then added to the Celution System where it was washed with a Lactated Ringers solution using gravity sedimentation/floatation. The concentrated ADRCs were then added to the washed graft tissue in the system and mixed to create the ADRC-enriched fat graft. The washed fat graft was found to contain 35% water evenly dispersed through the graft material and termed “wet graft.” Following the completion of tissue processing, the ADRC-enriched fat graft was aseptically transferred to the sterile field using 10 cm^3^ syringes.

The ADCRs were prepared successfully from each patient. We did not count the average of ADCRs after cell processing because it was not indicated in the initial design; thus, it was not approved by the ethical commission. But we estimated our results in a previous study leaded by us, in a similar and homogenous population [14]; ADRC yield after cell processing was 240,000 cells/g. Cell viability before injection was 86.6% ± 4.9%. The phenotypic characteristics of ADRCs from the SVF were analyzed by flow cytometry in a subset of 15 sequentially enrolled patients (CD34: 70.4% (range 66.5–73.3), CD45: 21.9% (range 17.3–26.0), CD184: 13.8% (range 6.9–17.1), vascular endothelial growth factor receptors: 10.8% (range 6.7–17.3), CD31: 10.3% (range 8.7–14.5), CD71: 2.8% (range 1.3–5.7), and CD105: 1.7% (range 0.6–2.6)).

In group II, the harvested adipose tissue was processed by a centrifuge with a sterilizable central rotor and sleeves that hold 10 cm^3^ syringes. The centrifugation speed was 3000 revolutions/minute for 3 minutes. This separated the denser components from the less-dense components to create multiple layers. The upper level was primarily made up of oil. The middle portion was made up of potentially viable parcels of fatty tissue, and the lowest, most-dense level, was made up of blood, water, and lidocaine. The central layer was used as an AF graft which was introduced in a 10 cm^3^ syringe that was placed in a sterile mobile platform in order to avoid more decantation and adherence of the fat graft to the walls of the syringe. This procedure was done in a surgical laboratory located in the proximity of the operation room.

## 3. Clinical Assessments

General assessments included medical history and physical examination and hematologic analysis. Functional assessments included VHI-10 scoring, laryngoscopic evaluation, and biomechanical vocal fold evaluation.

### 3.1. Laryngoscopic Evaluation of Injected Vocal Folds

Laryngoscopies were practiced 15 days before the surgery and after it: 30 and 180 days after the operation and every 180 days, using a 4.0 mm, 30° rigid endoscope (Richards). Laryngeal images were taken using a digital camera (E4500; Nikon, Tokyo, Japan) attached to the rigid endoscope. Data recorded made a description of the aspect of the laryngeal mucosa, the affected VF, its position, description of the GG, and a description of the postoperative compensation.

**Figure 1:**
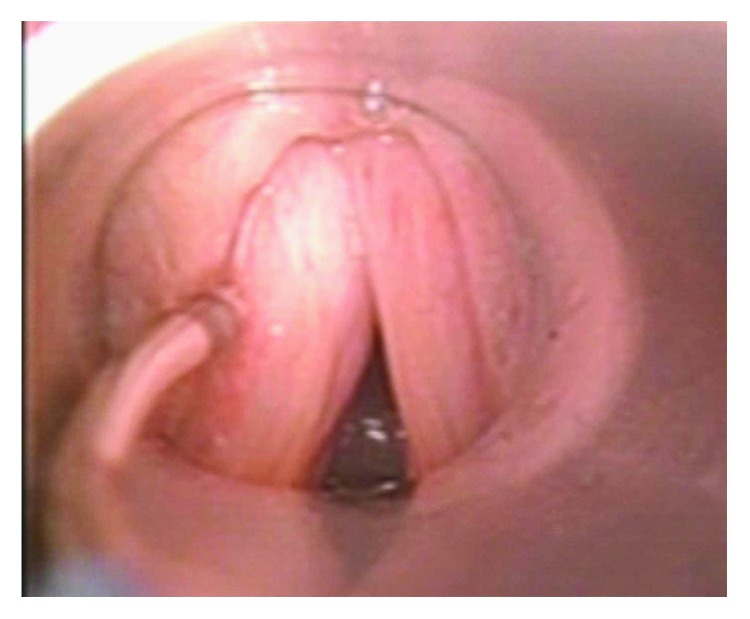
Endoscopic captures of the vocal folds in the patient FS4 (group I) before, during, and after injection (pictures captured from endoscopic video).

In Figure 1, the aspect of the glottal gap of a patient presenting unilateral left vocal fold paralysis is shown, during infiltration.

### 3.2. Quality of Life Scoring

Patient satisfaction with overall voice, bronchi-aspiration, and treatment results were assessed following the test Voice Handicap Index (VHI-30) [15]. The VHI-30 has been shown to be a valid and reliable instrument for assessing self-perceived handicap associated with dysphonia. The original VHI-30 was translated into Spanish and validated by the Phoniatry Committee of the Spanish Society for Otorhinolaryngology (SEORL) [16, 17]. Patients with the highest self-perceived dysphonia scores should get the highest scores on the VHI questionnaires, ranging from 0 to 4. The questionnaires were filled 1 month before the surgery and at the postoperative day 180. Later on, they were filled every 180 days. Only the answers to the functional part of the questionnaire were used in the present study.

### 3.3. Biomechanical Characterization of Voice

One of the reference techniques used in the evaluation of voice quality after treatment was the estimation of vocal fold biomechanical parameters. One of the most relevant biomechanical parameters evaluated was the vocal fold stiffness, which may be estimated separately on the body (*musculus vocalis*) and the cover (*lamina propria*). The estimation of the biomechanical stress acting on *musculus vocalis* requires the reconstruction of the glottal source from a voiced segment of speech (preferably an open vowel) by the inversion of the vocal tract by a lattice adaptive filter [18]. Accurate spectral domain techniques allow the estimation of a set of biomechanical parameters associated to a 2-mass model of the vocal folds from the glottal source spectral density; this set of features, describing the viscoelastic vibration of the vocal folds in mechanical terms, is obtained from voice using a mathematical technique which is known as vocal tract inversion by LPC (linear predictive coding) [19]. As a result, an estimate of the vocal fold body mechanical stiffness is produced for each phonation cycle.

**Figure 2:**
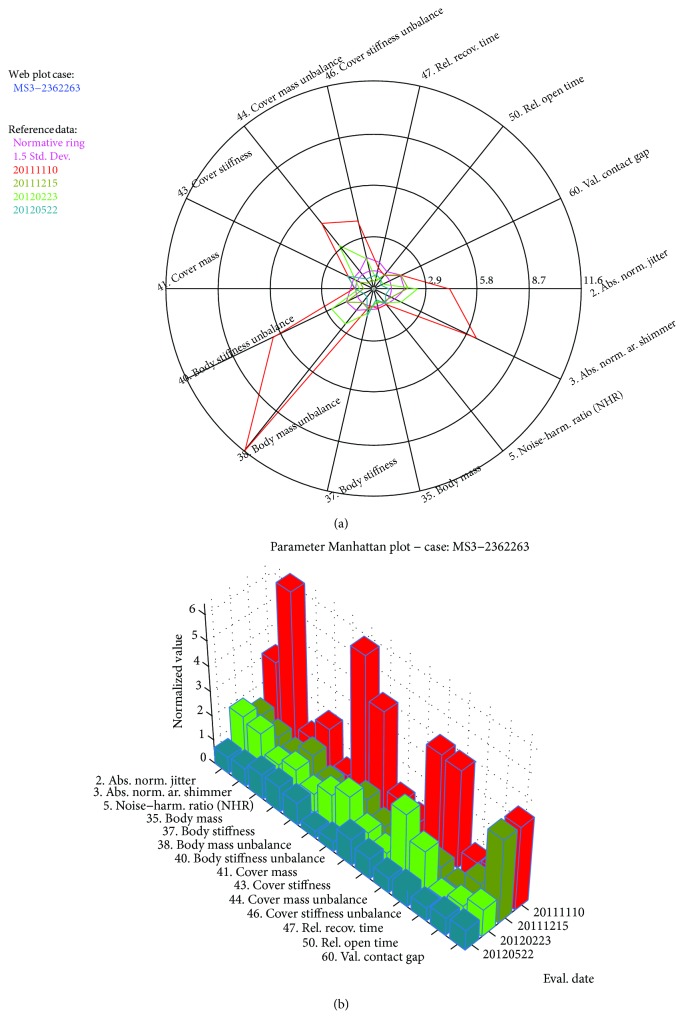
(a) Web plot of the longitudinal study. The evolution of the 14 parameters selected is shown chronologically from red (pre) to dark blue (post). Each normalized feature must be read on the intersection of each polygon with the corresponding feature radius. Clearly, features 2, 3, 35, 38, 40, 44, 46, and 60 are beyond the normality limits. (b) Manhattan skyline of the same study. Each feature from the four session recordings is presented chronologically from red to dark blue. The different features are now presented as polyhedral columns, the height of the column giving the normalized value of the feature relative to the population mean.

BioMet®Phon (version 9.2) is a set of software applications developed for the biomechanical characterization of VF in different fields as voice quality evaluation in laryngology, speech therapy and rehabilitation, education of the singing voice, forensic voice analysis, or emotional detection in voice [13]. The software allows the handling of a small patient's database. Once a patient is selected, either a new recording may be obtained and analyzed or an old one may be processed. The results of the longitudinal evaluation of each parameter for the four different examination instants for a case may be seen plotted in Figures 2(a) and 2(b).

### 3.4. Evaluation of Voice Quality Improvement by Likelihood

The modification in voice quality can be expressed numerically using likelihood estimations. The analysis is based in feature vectors **x**_m/f,*i*_ estimated from sustained vowel/a/phonations, which constitute a set of observations for each speaker given by matrices **X**_m_ and **X**_f_ (m: male set; f: female set). Each vector **x**_m/f,*i*_ from speaker *i* integrating these matrices stores the average estimates for each one of the 14 features described in Table 1.

In evaluating the improvement in voice quality as a functional result from surgery, the proposed methodology is based on the use of the log likelihood improvement ratios (LLIR), a metrics founded on alternative hypothesis testing [20], originally developed for its use in forensic speech evidence matching [21]. In the present case, two alternative hypotheses are considered:

- H0: the observations vector **x**_*i*_, integrated by the 14 features, has been generated by a parametric distribution Γ_N_ from a normative speaker set (hypothesis of normophony).
- H1: the observations vector **x**_*i*_ has not been generated from the normative distribution Γ_N_ (hypothesis of dysphonia).

A specific test based on LLIRs assumes that two observations **x**_A_ and **x**_P_ from the patient at different time instants are available. The pretreatment observation **x**_A_ is supposedly the earliest one (A: ante), and the posttreatment observation **x**_P_ corresponds to the latter one (P: post), relative to the treatment whose effects on the phonation functional improvement are going to be assessed. The log likelihood improvement ratio (LLIR) is defined as the natural logarithm of the conditioned probabilities of both pre- (A) and post- (P) observations relative to H0 (normophony).

If the probability of **x**_P_ being generated by the normative distribution Γ_N_ is larger than the probability of **x**_A_ being generated by the same distribution, it seems that the posttreatment evaluation fulfils better H0 than the pretreatment evaluation; thus, an improvement in phonation has likely occurred which could be attributed to treatment success. On the contrary, the results could be attributed to worsening phonation conditions. It is clear that phonation improvements will produce positive LLIRs and phonation worsening will produce negative LLIRs.

## 4. Results

### 4.1. Test Dates

The reporting period ranged from October 2011 to February 2014. In this clinical trial, sixteen patients were screened. There were two screening failures, and 14 patients underwent treatment and follow-up. During the reference period, no deaths were reported or major complications related to the technique.

### 4.2. Age, Gender, and Pathology

The relation of patients included in the study is given in Table 2, indicating their age and gender, the primary diagnosis they received, the primary treatment applied, the collateral consequences in laryngeal conditions, and their treatment with ADRC or CAF.

The final distribution of patients was as follows:

- The overall mean age is 52.49 years with a standard deviation of 16.60 years.
- Group I (ADRC) has 7 patients (3 males and 4 females) with a mean age of 52.28 years and a standard deviation of 20.95 years.
- Group II (CAF) has 7 patients (3 males and 4 females) with a mean age of 52.71 and a standard deviation of 12.59 years.
- The female set showed a mean age of 56.44 and a standard deviation of 18.30 years.
- The male set showed a mean age of 51.00 years and a standard deviation of 15.61 years.

### 4.3. Fat Processing and Injection

The mean volume of autologous fat harvested was 182.51 ± 12.57 cm^3^ (total average), 197.82 ± 15.23 cm^3^ (average for the group treated with ADCRs), and 167.10 ± 33.02 cm^3^ (average for the group receiving CAF). The total amount of fat (both groups) that was injected in deep into the paralyzed side of the thyroarytenoid muscle was 1.79 ± 0.29 cm^3^. In the group treated with ADCRs, the mean infiltrated volume of processed fat was 1.65 ± 0.56. In the group receiving CAF, the mean infiltrated volume was 1.93 ± 0.98 cm^3^.

### 4.4. Satisfaction Scores

As the VHI-10 test is formulated with ten questions, each accepting a possible answer between 0 and 4, and the maximum possible value would be 40 (strongly disappointing voice). Smaller values indicate subjective appreciations close to normality. The results of the tests before treatment (pre) and after treatment (post) are summarized in Table 3.

The overall preoperative mean score taking into account for all patients is 24.21 ± 8.28. The overall postoperative mean score for all patients is 6.71 ± 6.75. The partial preoperative mean score for group I is 21.14 ± 3.58. The partial postoperative mean score for group I is 3.14 ± 3.53. The partial preoperative mean score for group II is 27.29 ± 10.66. The partial postoperative mean score in group II is 10.29 ± 7.52.

**Figure 3:**
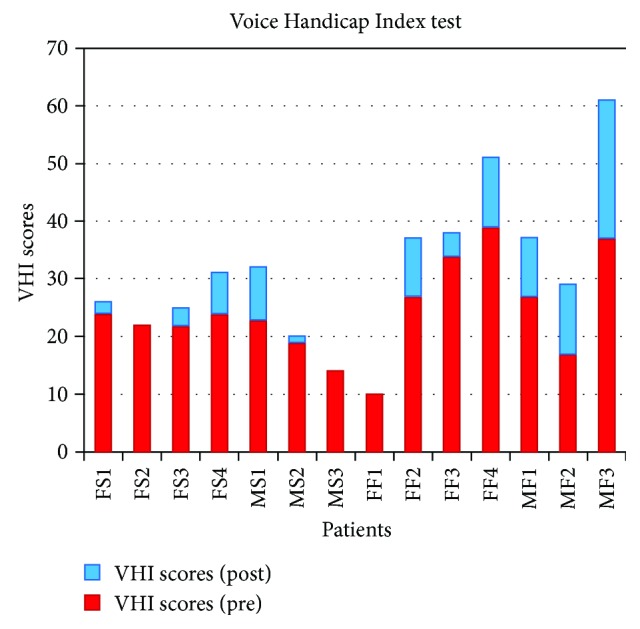
Evolution of the satisfaction scores for groups I (FS and MS) and II (FF and MF).

It may be seen that postoperative score means are less far apart from normality than preoperative means. Besides, the estimation error is much lower in the postoperative case, although certain differences may be appreciated between group I and group II.  Figure 3 shows the distribution of data of patients in both groups.

The figure shows the VHI score for each patient before (red) and after (blue) treatment. The VHI score is based on subjective opinions of the patient about different questions ranging from 0 to 4 (0—never, 1—almost never, 2—sometimes, 3—almost always, and 4—always). The smaller the score, the largest the satisfaction of the patients with respect to their voice is. It may be seen that in general, patients in group I (treated with stem cells and autologous fat) manifest smaller VHI pretreatment scores than those in group II (treated only with autologous fat), and the respective means being, respectively, 21.14 and 27.29. It is important to evaluate if this difference is significant enough so as to condition posttreatment results, thus masking the possible differences in the treatment results that are the objective of the study. For such, two tests have been used: Wilcoxon rank sum and Student's *t*-test. The results of both tests are shown in Table 4.

It may be seen that both the Wilcoxon and the Student *t*-tests (GI pre versus GII pre) cannot reject the null hypothesis regarding the statistical significance of group I and group II VHI tests. Therefore, it cannot be said that both distributions are significantly different, thus availing the possibility of comparing both sets of patients although the means of their pretreatment VHI scores are different. On the contrary, both tests reject the null hypothesis at 5% significance level (GI post versus GII post) when the posttreatment results for both groups are compared. This result is of certain relevance, as it states that the patient's self-perception of posttreatment improvement is larger when ADRCs are included when only fat is used. The results of comparing GI pre versus GI post reveal a real functional improvement, which is statistically relevant given the very small *p* values obtained, that allow rejecting the null hypothesis. The rejection of the null hypothesis is also significant for group GII, although not as strong as for GI (9.324*e* − 3 and 4.821*e* − 3). We can state that pretreatment tests from GI and GII show that these groups are acceptable for comparison, that posttreatment tests reveal that both groups behave differently after treatment, and also that both groups show statistically significant differences in self-perceived phonation improvements, although the significance is larger for GI than for GII under both tests.

### 4.5. Evaluations from Direct Laryngoscopy

The description of the glottal gap conditions observed in patients before and after the treatment is given in Table 5 from laryngoscopy inspections prior and 180 days after surgery.

The only remarkable incidence reported was from a patient treated by ADRCs showing an initial congestion of the treated VF, presenting an irregular closure pattern, with a fusiform gap in the laryngoscopy taken 180 days after treatment (MS3).

Two patients of the CAF group showed incomplete coaptations of the GG (FF1 and MF2), presenting a partial closure pattern.

### 4.6. Voice Quality Improvement

Table 6 gives the dates of the pretreatment and posttreatment voice quality evaluations.

The results of evaluating the pre- and posttreatment voice quality conditions on each of the patients from the likelihood ratios are given in Table 7.

The first column from the left gives the patient's summary code (F: female; M: male; S: stem cell + fat; F: only fat). The second column explains if the implant transferred ADRCs in autologous fat (ADRC) or not (CAF). The third column gives the log of the probability for a feature template **x**_A_ extracted from phonation before treatment matching the normative feature distribution Γ_N_. The fourth column gives the log of the probability for the feature template **x**_P_ extracted from phonation after treatment matching the same normative feature distribution. The fifth column gives the log likelihood improvement ratio. The sixth and seventh columns give the Voice Handicap Index score before (A) and after (P) treatment. The eighth and rightmost column gives the difference between the VHI scores (before and after). The bottom row gives the correlation coefficients between the before treatment probability (log{Pr(**x**_A_∣**Γ**_N_)}) and VHI score the after treatment probability (log{Pr(**x**_P_∣**Γ**_N_)}) and VHI score and the *λ*(**x**_*A*_ | **x**_*P*_) and the difference between the pre- and post-VHI scores (VHI(A) − VHI(P)).

In general, eight out of fourteen patients experience LLIRs over 100, which is assumed to be the reasonable threshold for claiming a substantial qualitative improvement. Four other patients experience slight improvements (MS2, MS3, FF3, and FF4). Two of them experienced a slight setback (FS2 and FS4). The three largest improvements (MS1, FS3, and FS1) correspond to cases treated with ADRC, compared to three smaller ones corresponding to CAF (MF2, FF2, and MF3). In general, ADRC cases behave more irregularly than CAF ones: either produces the largest improvements or very modest ones. Two cases treated with ADRC show moderate improvements (MS2 and MS3) versus four ones treated with CAF (MF1, FF1, FF3, and FF4). Voice quality improvements, in cases where present, are mainly due to a reduction in the unbalance of vocal fold biomechanics (parameters 40, 42, 44, and 46). This can be observed both for ADRC and CAF cases. Accordingly, it can be said that both techniques can be considered successful under the point of view of voice quality analysis.

Another important analysis is the correlation between logarithmic probability indices of separation from the normophonic model for each case (pre- and posttreatment), and the corresponding VHI scores. The correlation coefficients between the different groups considered are shown in Table 8.

At this point, it must be emphasized that the correlation between the pretreatment logarithmic probability measuring the separation of phonation from the normophonic model log{Pr(**x**_A_∣**Γ**_N_)} and the corresponding VHI score for the whole set of cases (group I and group II) is of −0.46, indicating that log scores are negative whereas VHI ones are positive and that the degree of relationship is reasonable between both measurements. The relationship is similar when posttreatment measurements are correlated (−0.42). The relationship between the LLIR and the pre- and posttreatment score differences is also similar (0.42), showing similar signs in this case. The situation reflected when both groups are separated is rather different. For group I (ADRC), the correlation coefficients are substantially larger (−0.63, −0.77, and 0.54, resp.). For group II (GAF), the correlation between probabilities of normophony and VHI scores is a bit worse (−0.49, −0.40, and 0.33). These results may indicate a better consistence between voice evaluation quality indices and VHI scores for the ADRC group.

At this point, two special cases require a further study, these being FS2 and FS4 (both patients treated with ADRC). In these cases, it must be mentioned that the sets of features used in the analysis of voice quality did not reveal substantial changes between pre- and posttreatment conditions. Besides, simple listening of their phonation in pre-and posttreatment conditions did not reveal important perceptual distortion (GRBAS was evaluated as mild in both cases). Nevertheless, the VHI revealed that both patients were concerned about the state of their voice, as expressed in Table 9.

The initial conditions of both patients are almost the same, the scores being relatively similar. Both complain mainly about problems with understanding by others and clarity of phonation. The final conditions express a reasonably high acceptance of the improvements experienced. Inexplicably, the initial conditions are not reflected by the pretreatment voice quality analysis. A reasonable hypothesis is that probably the set of features used in the study cannot detect the distortions perceived in the subjective autotest implied in VHI and that some other complementary features should be included in the study.

It must be mentioned that given the size of the case study described, statistical relevance of the results is very limited. Nevertheless, a similar evaluation as the one carried out for the VHI results expressed in Table 4 should be of the same interest. In this case, it must be mentioned that given the dispersion of logarithmic probabilities as given in Table 7, parametric tests are not the best choice. Instead, Wilcoxon rank sum has been used. The evaluation of statistical significance of voice quality analysis is given in Table 10.

As before, the comparison of ADRC versus GAF pretreatment voice quality analysis (GI pre versus GII pre) cannot reject the null hypothesis under a 5% significant level (*p* value = 0.901); therefore, to a certain extent, pretreatment voice quality analysis from both groups can be compared. The same conclusion can be derived from posttreatment results (GI post versus GII post, *p* value = 0.535). When comparing pre- and posttreatment results for the ADRC group analysis (GI pre versus GI post), the *p* value for rejecting of the null hypothesis is near the limit (0.053). The rejection of the null hypothesis is clearly possible for the GAF group analysis (GII pre versus GII post) a *p* value well below the limit (0.007). As it must be reminded, the null hypothesis establishes that if the pre- and posttreatment distributions would be equivalent, there would be no significant differentiation between pre- and posttreatment results. This is clearly true for the GAF group and almost in the limit for the ADRC group. Plausibly, the worse behavior of this last group may be caused by cases FS2 and FS4.

These results have to be seen under the exploratory nature of the study, considered a pilot to initiate further research in the topic. Having stated these considerations, it may be said that both procedures, on the one hand CAF, and on the other hand ADRC, procure important improvements in most of the cases as far as voice quality is concerned, under an objective basis.

### 4.7. Adverse Events

During this period, two adverse events have been reported in one patient (septic shock and glottis oedema). It occurred in the experimental arm but related to the intubation procedure and not to the experimental intervention reported. Both cases were removed from the study.

## 5. Discussion

### 5.1. General Considerations

VF paralysis can be unilateral or bilateral. When it is bilateral and during the adduction phase, tracheotomy is the treatment of choice; but when it appears during the abduction, the patient will present a GG, resulting in hoarseness of voice, aspiration of nutrients, dyspnea, and the impossibility of exerting normal body effort, thus affecting the patient's quality of life [1].

Surgery of UVFP consists basically in two techniques: open surgery on the laryngeal box and injection laryngoplasty with autologous or nonautologous materials [2]. Open procedures require a major surgical operation, and conditions after a follow-up at six months [22] may be similar to the initial situation.

In relation to the second option, surgeons have attempted to repair aerodynamic incompetence generated by GG augmenting the volume of the VF by means of injection laryngoplasty. VF consist of a pliable tissue layer known as lamina propria, which is sandwiched between epithelium and skeletal muscle. This is a loose connective tissue containing elastin, collagen, and fibroblast-like cells. VF geometry is critical to develop a proper function; thus, a selected scaffold material should provide lasting levels of augmentation while allowing for a new tissue formation. In order to this, the ideal filler must be biocompatible and not reactive with the host, long lasting, and easy to obtain. The most used fillers in the clinical practice are collagen, hyaluronic acid, and autologous fat [23]; although these materials resulted in VF improvements, postimplantation resorption or compactation has limited the long-term success.

### 5.2. Fat Tissue and Vocal Fold Laryngoplasty

Among autologous tissues for refilling, fat is currently one of the most appreciated resources for regenerative medicine. It is a readily available tissue that presents most of the characteristics required to be the ideal filler. It may be generously injected and can be easily harvested in the operating room under sterile conditions. Once more, the problem of using autologous fat injection for laryngoplasty is whether the injected fat maintains the graft volume, which may be dependent on fat preparation techniques; in fact, many authors suggest fat overinjection due to this variability [24–26]. Complications reported after AF refilling are another negative aspect, which by fortune is not frequent. In a retrospective work on 88 patients with a mean follow-up of 20.2 months, laryngeal complications occurred in 4.5% of patients, including 3.4% cases of overinjections that lead to poor voice quality and the formation of granuloma (1.1%). Overinjections were managed using cordotomy with fat removal [27]. Thus, the fate of injected fat continues to be debated as its survival seems to be highly variable.

In a similar study [28], CAF has been successfully used for VF refilling; 14 patients with breathy dysphonia secondary to laryngeal hemiplegia and patients presenting anatomical defects (7 cases, resp.) underwent vocal fold lipoinjection with CAF only. The fat cell layer was injected into the vocal muscle and patients underwent pre- and postoperative videolaryngostroboscopy and Voice Handicap Index (VHI) self-assessment. Voice quality improved soon after surgery like in our patients and remained stable over more than 10 months. Interestingly, the results were better in the patients with paralytic dysphonia. Then, CAF is an accepted method to improve VF volume in order to treat the GG insufficiency, showing similar results to ours.

### 5.3. Stem Cells and Vocal Folds

Some of the potential treatment modalities reported for vocal fold scar include injection of mesenchymal stem cells (MSCs), adipose-derived stem cells (ADSCs), autologous fibroblasts, and potential derivatives of human embryonic stem cells [29].

ADSCs and MSCs have been shown to increase angiogenesis to ischemic tissue since these cells seem to excrete substantial quantities of angiogenic growth factors [30, 31].

Lately, it has been described that human vocal fold fibroblasts (hVFF) isolated from the *lamina propria* meet the criteria established to define MSCs and are functionally similar to MSCs derived from bone marrow and adipose tissue. hVFF have the same potential as MSCs, and Hanson et al. proposed that vocal fold fibroblasts are MSCs residing in the *lamina propria* [32].

Human adipose-derived stem cells (hADSCs) have been used in the refilling of the VF in different experimental models [33]. These cells showed to be a good source for vocal fold tissue engineering; they have the ability for promoting injured vocal folds and also play an important role in vocal fold reparation and regeneration.

In a model of injured VF in rats, refilling with ADSCs versus bone-marrow stem cells showed that one month after surgery, there were increased levels of hyaluronic acid and decreased levels of collagen deposition in VF of both groups. It was suggested that regenerative effects of both types of stem cells were similar, but adipose cells might present a better recovery of hyaluronic acid and superior antifibrotic effect [34]. Interestingly, Kim et al. demonstrated that hydrogel-containing hADSCs injected into the VF of rabbits improved VF healing. Endoscopic and functional evaluations performed one and three months after injury revealed that this technique was promising for prolonging the retention time of stem cells in VF [35].

Nevertheless, regarding the results reported in the present study, the effects of the treatment seem to be partially long lasting in both groups, while satisfaction improved more significantly in group I in comparison to group II. It has been described that fibrosis stiffens muscle decreasing engraftment efficacy and altering cell fate. ADSC injection into fibrotic muscles showed this effects, but myotubes derived from ADSC when replanted onto a stiff matrix maintained their fused state, which could explain the scores of satisfaction in group I [36].

It is accepted that seeding cells is an important part of tissue engineering but it is still difficult to find the most suitable seed cell in the larynx, which presents a limited space. We consider that perhaps the main existing difficulty lies in that cells are not able to form the original structure in vivo after planting, which may also explain the reduction of volume after promising initial results, especially if there is fibrosis of the *lamina propria* and/or of the vocal muscle.

Taking into account the extensive history of injectable biomaterials in laryngeal surgery, a major focus of regenerative therapies must be the development that shall control in vivo residence time and elastic properties of the native tissues.

Isolated adipose stem cells conditioned in scaffolds or foams, hydrogel matrix [37], or decellularized adipose tissue (DAT) seem to induce a strong angiogenic response demonstrating soft tissue regeneration [38]. Though DAT may be obtained by tissue printing [39], there are still limitations which insert these tridimensional structures into VF.

Adipose-derived regenerative cells (ADRCs) seem to be a promising alternative to autologous fat therapy. During the last decade, cellular therapy based on these cells has emerged as a suitable method to face some clinical problems like breast reconstruction or myocardial ischemia [11, 14]. In this work, we wanted to evaluate the safety and use of autologous ADRCs for increasing the volume in UVFP leading to GG insufficiency. Therefore, it is expected that the development of a simple and stable source of differentiated cells as seed cells would be useful for VF wound healing.

ADRCs, frequently referred in the literature as stromal vascular fraction (SVF), are a heterogeneous cell population most commonly derived from the manipulation of adipose tissue through enzymatic digestion, removal of adipocytes (on the basis of their inherent buoyancy), washing, and concentration by centrifugation. ADRCs contain not only the typical supportive stroma found in vivo to anchor and nurture adipocytes but also cells from the hematopoietic system including those that are both normal residents in adipose tissue and those recruited during adipose tissue collection by liposuction. The heterogeneous nature of ADRCs makes the characterization of cell identity and purity challenging. Despite this, progress has been made using conventional flow cytometry methodologies [40] to identify major cell subpopulations of ADRCs and subpopulations with the potential to contribute to efficacy.

ADRCs also contain a substantial number of endothelial progenitor cells (EPCs) and adipose stromal cells, both of which express the CD34 marker. EPCs play a role in therapeutic vasculogenesis and can increase tissue perfusion and improve wound healing [8, 9]. These properties may favor the engrafting of the enriched autologous fat with ADRCs into the vocal fold.

The CD34+ population is also known to harbor the adipose-derived stem cells (ADSC). ADSCs are multipotent cells capable of differentiating into multiple lineages, such as adipocytes, osteoblasts, chondrocytes, myocytes, endothelial cells, hepatocytes, and neurons in vitro, given the appropriate specific conditions and stimulating factors, and may be direct contributors to new vascular tissue as well [41, 42].

### 5.4. Functional Results

At this point, it is of most relevance to pose the main questions related with the use of grafting techniques in vocal fold refill, which are the following:

- If using CAF or ADRC grafting can be considered efficient and safe methods in the restoration of the phonation function
- If the ADRC method improves the phonation function over the CAF method

The answer to these questions is a difficult one under the light shed by the results reported in the present study, due to its size limitations. Nevertheless, it may be said that

- both methods seem to preserve, and in most cases, improve the phonation function, reestablishing the equilibrium between both vocal folds, initially unbalanced. This is true in all cases, even in the ones with the lowest LLIR: the improvements are produced by a reduction in the vocal fold unbalance parameters (body and/or cover). Unbalance being one of the causes for rough voice, both techniques reduce this perceptual quality. The cases experiencing a small setback (FS2 and FS4) show a slight worsening in vocal fold body and cover stiffness, possibly as a reaction to the treatment (parameters 37 and 43). But given the case that both parameters do not separate much from the normophonic limits, it does not seem that this setback could be significant, possibly being amplified by the sensitivity of the likelihood estimation methodology used in the study. On these bases, it cannot be said that ADRC is not so safe than CAF;
- when comparing the restoring capability of both methods, it must be said that as far as the phonation quality is concerned, the best results are obtained with ADRC than with CAF. Nevertheless, the worst results are obtained also with ADRC;
- the results are not homogeneous. Some cases experience a strong improvement whereas others do not. This happens in cases where phonation quality is not very bad in pretreatment conditions. It may be due to the capability of the selected features to represent phonation quality deterioration, this fact requiring further study;
- the statistical significance of the study is not relevant, due to the number of cases included, and their despair age, and pathological conditions. Nevertheless, the results encourage to extend the study to gain better insight into the possibilities of voice quality evaluation in assessing functional success;
- a very important factor to be contemplated in future studies is the influence of the time left to assess functionality after treatment, especially for the ADRC technique, as it is expected that in cases where the grafts are successful, the improvement should be larger than in CAF. Unfortunately, the distance between observation intervals did not contemplate this factor in the present study.

In general, it seems that the effectiveness of treatments is influenced neither by age nor by gender. The largest improvements correspond to two of the elder patients, male and female (MS1 and FS3), both treated with ADRC. Three of the patients with larger improvement indices are males (MS1, MF2, and MF3), whereas three others are females (FS1, FS3, and FF2). Regarding cases showing moderate improvement rates, it may be said that all of them present pre- and posttreatment recordings which are only slightly dysphonic; therefore, the expected improvement is not large.

Finally, it must be mentioned that none of the patients developed tumors at the VF and indeed not systemic tumorigenesis after treatment with ADRCs.

## 6. Conclusions

The FIBHGM-ECNC007-2010 clinical trial has been the first study to assess changes and clinical outcomes in the VF defect in a patient after refilling with AF enriched with ADRCs.

This prospective trial demonstrates the safety and efficacy of the treatment of UVFP (glottal gap) defects utilizing ADRC-enriched fat grafts.

The ADRC procedure is feasible and allows a direct comparison with CAF.

The satisfaction scores for both groups show a subjective perception of general improvement in their laryngeal use and voice quality. Satisfaction score improvements are larger for group I than for group II.

Voice quality ratios show an objective improvement for both groups with two exceptions for group I, besides the cases with larger improvement ratios were found in group I, although improvement ratios are fairly acceptable for group II as well.

Both methods have shown to be efficient in restoring the phonation function when examined using distortion, biomechanical, and gap features. No substantial differences have been found in this respect between both methods.

Apparently, neither age nor gender factors influenced the results achieved by both methods.

None of the patients showed important negative side effects in their larynx posttreatment conditions.

## Figures and Tables

**Table 1:** Parameter description.

Parameter number	Description
2. Jitter	Variation of period between two consecutive glottal cycles relative to its mean
3. Shimmer	Variation of glottal source average between two consecutive glottal cycles relative to its mean
5. NHR	Ratio between the energy of the turbulent part of the glottal source power spectrum relative to its total energy
35. Body mass	Dynamic component of the inertial part of the vocal fold body (10^−3^ g)
37. Body stiffness	Elastic force distributed in length over the musculus vocalis (10^−3^ N/m)
38. Body mass unbalance	Variation of parameter 35 between two consecutive glottal cycles relative to its mean
40. Body stiffness unbalance	Variation of parameter 37 between two consecutive glottal cycles relative to its mean
41. Cover mass	Dynamic component of the inertial part of the vocal fold cover (10^−3^ g)
43. Cover stiffness	Elastic force distributed in length over the lamina propria (10^−3^ N/m)
44. Cover mass unbalance	Variation of parameter 41 between two consecutive glottal cycles relative to its mean
46. Cover stiffness unbalance	Variation of parameter 43 between two consecutive glottal cycles relative to its mean
47. Relative recovery time	Time interval from the maximum flow declination rate to the end of the glottal source quiescent value
50. Relative open time	Time interval from the maximum flow declination rate to the starting point of the open phase
60. Value of contact gap	Ratio between the air escape during defective contact episodes and air escape during the open phase

**Table 2:** List of patients treated in the study.

Code	Gender	Age	Diagnostic	Treatment consequence	Implant
FS1	F	47	Thymus thickening	Recurrent laryngeal paralysis	ADRC
FS2	F	36	Thyroid papillary carcinoma	VFP after total thyroidectomy	ADRC
FS3	F	84	Idiopathic recurrent laryngeal paralysis		ADRC
FS4	F	30	Acoustic nerve neurinoma	Facial and VF paralysis after primary surgery	ADRC
MS1	M	79	Esophageal adenocarcinoma	VFP after esophagectomy	ADRC
MS2	M	48	CNX schwannoma	VFP after primary surgery	ADRC
MS3	M	42	Paraganglioma	VFP after primary surgery	ADRC
FF1	F	52	Paraganglioma	VFP after primary surgery	CAF
FF2	F	52	Pontocerebellar epidermoid carcinoma	VFP after primary surgery	CAF
FF3	F	52	Paraganglioma	VFP after primary surgery	CAF
FF4	F	76	Multinodular goiter	VFP after primary surgery	CAF
MF1	M	55	Thymoma	VFP after primary surgery	CAF
MF2	M	49	Cholesteatoma and paraganglioma	VFP after primary surgery	CAF
MF3	M	33	Carotid and jugular paraganglioma	VFP after primary surgery	CAF

**Table 3:** Statistical description of VHI test results.

	Global	GI & GII		GI		GII	
		Pre	Post	Pre	Post	Pre	Post
Means	15.46	24.21	6.71	21.14	3.14	27.29	10.29
Std. Dev.	11.59	8.28	6.75	3.58	3.53	10.66	7.52
Conf. Int.	4.49	4.78	3.90	3.31	3.27	9.86	6.96

**Table 4:** Significance of VHI test results (*p* values).

	GI pre versus GII pre	GI post versus GII post	GI pre versus GI post	GII pre versus GII post
Wilcoxon rank sum	0.156	0.032	5.827*e* – 4	9.324*e* – 3
Student *t*-test	0.174	0.042	6.424*e* − 7	4.821*e* − 3

**Table 5:** Pretreatment and posttreatment descriptions from laryngoscopy.

Patient	Preoperative laryngoscopy	Postoperative laryngoscopy
(1) Group I (FS1)	Left VF paralysis; median position	Closure pattern
	Anteroposterior gap	2/3 anterior contact
	Normal mucosa	Good compensation
		Normal mucosa

(2) Group I (MS1)	Left VF paralysis; atrophy of left VF	Total closure pattern
	Anteroposterior gap	Good compensation
	Normal mucosa	

(3) Group I (MS2)	Right VF paralysis; lateral position of right VF	Total closure pattern
	Anteroposterior gap	Good compensation
	Normal mucosa	Normal mucosa

(4) Group I (MS3)	Atrophy of left VF	Irregular closure pattern
	Anteroposterior gap	Fusiform gap
	Normal mucosa	Normal mucosa after initial congestion
	No coaptation in phonation	

(5) Group I (FS2)	Left VF paralysis; paramedian position	Total closure pattern
	Anteroposterior gap; 1-2 mm gap in phonation	Good compensation
	Normal mucosa	Normal mucosa

(6) Group I (FS3)	Atrophy of left VF	Total closure pattern
	Anteroposterior incomplete gap	Good compensation
	Normal mucosa	Normal mucosa

(7) Group I (FS4)	Paralysis of the right VF	Good coaptation in anterior 2/3
	Anteroposterior incomplete gap	Physiologic posterior hiatus
	Small posterior hiatus	Normal mucosa
	Normal mucosa	

(1) Group II (FF1)	Left VF paralysis; partial compensation	Total closure pattern
	Anteroposterior gap	Good compensation
	Normal mucosa	Normal mucosa

(2) Group II (FF2)	Atrophy of left VF; intermedian position	Total closure pattern
	Anteroposterior gap	Good compensation
	Normal mucosa	Normal mucosa

(3) Group II (FF3)	Atrophy of left VF; fusiform hiatus	Total closure pattern
	Anteroposterior gap	Good compensation
	Normal mucosa	Normal mucosa

(4) Group II (MF2)	Atrophy of the right VF	Total closure pattern
	Anteroposterior incomplete gap	
	Normal mucosa	Good compensation
		Normal mucosa

(5) Group II (MF1)	Atrophy of left VF	Partial closure pattern
	Anteroposterior incomplete gap	Phonation in bands
	Small midposterior hiatus	Normal mucosa
	Normal mucosa	

(6) Group II (FF4)	Atrophy of left VF	Total closure pattern
	Anteroposterior incomplete gap	Good compensation
	No closure in phonation	Normal mucosa
	Normal mucosa	

(7) Group II (MF3)	Atrophy of left VF, presenting retraction in middle third and sulcus	Total closure pattern
	Anteroposterior gap	Good compensation
	No closure in phonation	Normal mucosa
	Normal mucosa	

**Table 6:** Pre- and posttreatment evaluation dates.

Patient's code	Implant method	Pretreatment evaluation date	Posttreatment evaluation date	Days between pre- and posttreatment evaluations
FS1	ADRC	15.09.2011	18.10.2012	399
FS2	ADRC	29.11.2011	28.02.2013	457
FS3	ADRC	14.06.2012	28.02.2013	259
FS4	ADRC	12.01.2012	27.02.2014	777
MS1	ADRC	22.03.2012	21.02.2013	336
MS2	ADRC	19.10.2011	15.01.2013	454
MS3	ADRC	10.11.2011	22.11.2012	378
FF1	CAF	21.06.2012	28.02.2013	252
FF2	CAF	04.10.2012	11.04.2013	189
FF3	CAF	28.10.2010	21.11.2012	755
FF4	CAF	24.03.2011	21.06.2012	455
MF1	CAF	17.11.2011	24.01.2013	434
MF2	CAF	24.07.2012	24.01.2013	184
MF3	CAF	20.04.2011	17.05.2012	393

**Table 7:** Voice quality improvement from likelihood ratios and VHI.

Patient's code	Implant method	log{Pr(**x**_A_∣**Γ**_N_)}	log{Pr(**x**_P_∣**Γ**_N_)}	*λ*(**x**_*A*_ | **x**_*P*_)	VHI(A)	VHI(P)	Diff
FS1	ADRC	−12628.24	−41.17	12587.07	24	2	22
FS2	ADRC	−34.91	−48.7	−13.79	22	0	22
FS3	ADRC	−15364.57	−7.44	15357.13	22	3	19
FS4	ADRC	−21.52	−32.03	−10.51	24	7	17
MS1	ADRC	−36276.17	−56.57	36219.6	23	9	14
MS2	ADRC	−46.09	−11.47	34.62	19	1	18
MS3	ADRC	−101.66	−5.05	96.6	14	0	14
FF1	CAF	−186.18	−20.44	165.74	10	0	10
FF2	CAF	−5041.86	−26.06	5015.79	27	10	17
FF3	CAF	−101.6	−15.48	86.12	34	4	30
FF4	CAF	−74.9	−16.77	58.13	39	12	27
MF1	CAF	−196.92	−3.47	193.45	27	10	17
MF2	CAF	−8844.82	−480.94	8363.89	17	12	5
MF3	CAF	−2436.14	−3.46	2432.69	37	24	13

**Table 8:** Correlation coefficients between voice quality estimates and VHI scores.

Correlation coefficient	log{Pr(**x**_A_∣**Γ**_N_)} versus VHIa	log{Pr(**x**_P_∣**Γ**_N_)} versus VHIp	*λ*(**x**_*A*_ | **x**_*P*_) versus VHIdiff
Group I + group II	−0.46	−0.42	0.42
Group I	−0.63	**−0.77**	0.54
Group II	−0.49	−0.40	033

**Table 9:** VHI results for cases FS2 and FS4.

Question	FS2a	FS2p	FS4a	FS4p
My voice makes it difficult for people to hear me	3	0	3	0
People have difficulty to understanding me in a noisy room	3	0	3	1
My voice difficulties restrict my personal and social life	3	0	3	1
I feel left out of conversations because of my voice	2	0	2	0
My voice problem causes me to lose income	0	0	0	0
I feel as though I have to strain to produce voice	2	0	2	1
The clarity of my voice is unpredictable	2	0	3	1
My voice problem upsets me	3	0	3	1
My voice makes me feel handicapped	2	0	2	0
People ask, what is wrong with your voice?	2	0	3	2
Totals	22	0	24	7

**Table 10:** Statistical significance of voice quality analysis (*p* values).

	GI pre versus GII pre	GI post versus GII post	GI pre versus GI post	GII pre versus GII post
Wilcoxon rank sum	0.901	0.535	0.053	0.007

## Data Availability

Liana De Plasencia is the clinical team leader of Dynamic with the following information: Tel. +34 91456 1105; Fax. +34 91456 1126; Mov. +34679763498; l.plasencia@dynasolutions.com.

## Abbreviations

ADRC: Adipose-derived regenerative stem cells
CAF: Centrifuged autologous fat
GG: Glottal gap
UVFP: Unilateral focal fold paralysis
VHI-30: Voice Handicap Index.
